# Uncovering latent trajectories of daily tinnitus symptoms through app-based monitoring during treatment

**DOI:** 10.1016/j.invent.2026.100943

**Published:** 2026-04-08

**Authors:** Milena Engelke, Berthold Langguth, Thomas Probst, Rüdiger Pryss, Stefan Schoisswohl, Jorge Piano Simões, Peter Ten Klooster, Beat Toedtli, Carsten Vogel, Winfried Schlee

**Affiliations:** a Department of Psychiatry and Psychotherapy, University of Regensburg, Regensburg, Germany; b Division of Psychotherapy, Department of Psychology, Paris Lodron University Salzburg, Salzburg, Austria; c Institute of Clinical Epidemiology and Biometry, University of Würzburg, Würzburg, Germany; d Department of Psychology, Health and Technology, University of Twente, Enschede, the Netherlands; e Institute for Information and Process Management, Eastern Switzerland University of Applied Sciences, St. Gallen, Switzerland

**Keywords:** Tinnitus, Treatment dynamics, Latent class analysis, Growth mixture modeling, Digital phenotyping, Ecological momentary assessment, Experience sampling

## Abstract

Tinnitus heterogeneity is well-documented across phenotypes, etiologies, risk factors, comorbidities and associated burden. However, variability in treatment response remains insufficiently explored and often masked by the group-level comparisons of clinical studies. Moreover, little is known about the temporal trajectories of symptoms during treatment. Longitudinal monitoring via smartphones using Ecological Momentary Assessment provides rich inter- and intraindividual data on fluctuations and trajectories of symptoms. In this study, we investigated whether individual 12-week trajectories of daily self-reported tinnitus symptoms during treatment could be meaningfully sub-grouped. 147 patients provided 9634 observations while undergoing single or combined applications of hearing aids, cognitive-behavioural therapy, structured counseling, and sound therapy. A four-class growth mixture model best fit the data. One class was characterized by an increase in tinnitus symptoms over time (18%), another showed stable symptom trajectories (40%), while the remaining two classes described symptom reductions with different onsets during treatment (early improvement: 20%; late improvement: 21%). The identified classes did not differ in baseline characteristics, indicating that this information could not be used to predict symptom trajectories. Additionally, all four classes were represented in nearly each treatment arm. Notably, retrospective patient-reported outcome measures (PROMs) did not consistently align with latent symptom trajectories. These results underscore the heterogeneity and non-linearity of symptomatic change both within and across treatment modalities. We propose that app-based trajectories reveal details about symptom improvement that cannot be seen in standard PROMs.

## Highlights

• Longitudinal monitoring provides rich data on symptom trajectories.
• Treatment response in tinnitus is a non-linear process.
• Treatment trajectories can be described by latent classes.

## 1 Introduction

Tinnitus is an auditory phantom condition which is perceived as a tonal or composite noise in the absence of an external sound source (Baguley et al., 2013). With a prevalence of 14% among adults and increasing rates with age, it is a common condition especially among the elderly (Jarach et al., 2022). Otological and non-otological factors could have been established as risk factors (Biswas et al., 2022). Tinnitus is characterized by its high heterogeneity in the percept itself, associated etiologies and comorbidites, in tinnitus-triggered distress as well as in response to treatment (Langguth, 2024).

While tinnitus heterogeneity has been well documented across perceptual characteristics, risk factors, comorbidities and severity (Langguth, 2024), variability in treatment response remains less understood. There is consensus that no single treatment is universally effective in reducing distress or loudness of the tinnitus percept (Cima et al., 2019; Schoisswohl et al., 2021). Despite that, clinical studies typically rely on group-level comparisons (Hoare et al., 2011). Although these studies are indispensable for proof of efficacy, this approach can obscure differential effects on individuals or subgroups and may contribute to inconsistent evidence observed across studies (Mckenna and Irwin, 2008; Hobson et al., 2012). At the same time, there is a subgroup of patients that benefit from treatment in most clinical tinnitus studies. Recent research aims to predict treatment response from extensive patient-level data assuming that outcomes vary with individual tinnitus characteristics and treatment fit (Bromis et al., 2022; Adcock et al., 2024; Shabestari et al., 2025; Niemann et al., 2020a; Rodrigo et al., 2022; Rodrigo et al., 2021). In this context, prescriptive predictors inform clinical practice about optimal treatment allocation for specific patients, while prognostic predictors indicate the likelihood of benefit regardless of treatment type. Yet there are no robust, generalizable results yet in tinnitus research due to diverse interventions and methodologies (Ivansic et al., 2022; Niemann et al., 2020b).

Simoes et al. crowdsourced data from 5017 tinnitus patients and found that individual characteristics had limited predictive value, but combined explained 16% of treatment outcome variance (Simoes et al., 2019). Niemann et al. identified and replicated four baseline phenotypes, ranging from low to high tinnitus distress with specific psychological or somatic burden (N = 1228 (Niemann et al., 2020c), N = 989 (Niemann et al., 2024)). They further described five treatment response subgroups, from deterioration to high improvement, whose distribution varied by baseline phenotype. Yet, predictive utility remained limited, highlighting the complexity of treatment trajectories (Niemann et al., 2024).

Most clinical studies rely on pre- and post-treatment assessments by patient-reported outcome measures (PROMs), however, tinnitus fluctuates over time depending on internal regulatory processes such as emotion, stress and circadian rhythm (Pryss et al., 2019; Engelke et al., 2023; Schlee et al., 2016). Further, continuously measuring treatment effects in daily life is rare, yet preliminary evidence describes diverse, non-linear tinnitus trajectories (Cuesta and Cobo, 2024; Seydel et al., 2010; De Ridder et al., 2025). Longitudinal research in neighboring mental-health fields has shown that patients can follow diverse therapeutic paths (Ulvenes et al., 2022; Saunders et al., 2019; Espel-Huynh et al., 2020). Capturing tinnitus symptom evolution throughout treatment at finer time scales might reveal diverse symptom trajectories as yet another layer of tinnitus heterogeneity.

In this study, we use intensive longitudinal data gathered in daily life by ecological momentary assessments (EMA) and growth mixture modeling to determine whether individual trajectories cluster into unobserved classes. This statistical technique identifies latent based on individual developmental trajectories and has been used to uncover heterogeneity in treatment courses in other mental health fields (Ulvenes et al., 2022; Saunders et al., 2019; Espel-Huynh et al., 2020). We then describe these classes by their course, size, and patient characteristics to advance understanding of heterogeneity in treatment response.

## 2 Methods

### 2.1 Study design

This exploratory analysis used data from a multicenter randomized clinical trial (RCT) on chronic tinnitus conducted between April 2021 and December 2022 in Germany, Greece, Spain, and Belgium (Schoisswohl et al., 2021). The study was approved by the local ethics committee at each site. Patients were randomized to one out of ten treatment arms which were group-based Cognitive Behavioural Therapy (CBT) focusing on tinnitus exposure, Hearing Aids (for patients with a hearing aid indication only), App-based Structured Counseling (SC), App-based Sound Therapy (ST) and the following combinations CBT + HA, CBT + SC, CBT + ST, HA + SC, HA + ST, and SC + ST. Detailed information on the study design can be obtained from the study protocol (Schoisswohl et al., 2021), the clinical effect of each treatment arm has been published (Schoisswohl et al., 2024).

### 2.2 Subjects

Patients primary complaint was chronic tinnitus (≥6 months), they were aged between 18 and 80 years, minimally distressed by their tinnitus (Tinnitus Handicap Inventory (Newman et al., 1996) [THI] ≥ 18), not cognitively impaired (Montreal Cognitive Assessment (Nasreddine et al., 2005) >22), and reported stable psychoactive medication for at least 30 days. We excluded patients with objective tinnitus, otosclerosis, acute ear infections, Meniere's disease, serious internal, neurological or psychiatric conditions, drug, medication or alcohol abuse within the last 12 weeks, severe hearing loss or one deaf ear, and if they had started another tinnitus treatment within the previous 12 weeks.

### 2.3 Study procedure

Patients underwent a baseline visit before they were randomized to a treatment arm. During the 12-week treatment phase, patients were prompted every evening at 7:30 pm by a dedicated smartphone app (Vogel et al., 2021) to fill out a daily EMA questionnaire. The remaining study visits took place after 6, 12, 36 and 48 weeks (in this study, we only use data from baseline and 12-week visit). During those visits, detailed clinical information regarding tinnitus, general health, comorbid diseases as well as audiological data were gathered on-site and online.

### 2.4 Data

We focused on the daily 10 EMA items assessing health-related and tinnitus symptoms (Table S1). Some questions refered to the momentary situation, others to the average over the day. A prior investigation identified changes in *tinnitus-related thoughts* to be best predictive for clinical improvement which was thus chosen as the main outcome (Engelke et al., 2025a). The distribution of the outcome over all patients and measurement points is shown in Fig. S1. The remaining variables predicting clinical improvement (Engelke et al., 2025a) were selected to be compared with the predicted trajectories of *tinnitus-related thoughts*. These are *jaw tension*, *momentary tinnitus loudness*, *daily tinnitus*, *maximum tinnitus loudness*, and *emotion*. Coding of *emotion* was reversed to *negative emotion* to standardize interpretation that higher scores indicate higher burden. These variables were chosen based on prior findings (Engelke et al., 2025a) and to reflect key dimensions of the tinnitus experience, including perceptual (momentary and maximum tinnitus loudness), affective (daily tinnitus distress, negative emotion), and somatic components (jaw tension). This selection was intended to capture both established and potentially underrepresented aspects of tinnitus-related burden (Engelke et al., 2025a; Michiels, 2023; Probst et al., 2016).

To describe baseline differences between patients of latent classes we analysed data of tinnitus case history (European School for Interdisciplinary Tinnitus Research Screening Questionnaire (Genitsaridi et al., 2019) [ESIT-SQ]), personality (Big Five Inventory-2 (Soto and John, 2017) [BFI-2]), tinnitus handicap (THI and Tinnitus Functional Index (Fackrell et al., 2016) [TFI]), depression (Patient Health Questionnaire-9 (Kroenke and Spitzer, 2002) [PHQ-9]), and hearing loss (average hearing threshold at the frequencies 500 Hz, 1 kHz, 2 kHz and 4 kHz [PTA-4]).

### 2.5 Data analysis

#### 2.5.1 Growth mixture models

In terms of data preparation, moving averages were calculated with a seven-day time window to smooth out fluctuations and weekly patterns thereby focusing on long-term trends. After visual inspection of the pre-processed outcome, one outlier was removed from the data.

To identify latent trajectories in *tinnitus-related thougths*, we estimated growth mixture models (Jung, 2008; Ram and Grimm, 2009) using the *hlme* function from the *lcmm* package in R. Fixed effects (overall and class-specific) included an intercept and a natural cubic spline function of time in days with three degrees of freedom to allow for non-linear trends over time. A random intercept accounted for subject-level variability within each class with variance constrained to be equal across classes. Models with progressively increasing number of latent classes (1−10) were estimated. For models with number of classes ≥2, an automatic grid search with 30 repetitions and 30 iterations was performed to prevent convergence at local maxima. Initialization values were provided from the 1-class model to improve stability and convergence. If the model did not converge, we increased the repetitions to 50 (this occurred only for the linear model with eight latent classes). To test whether a linear trend was more appropriate, we repeated the process with a linear function of time in days. We adhered to the Guidelines for Reporting on Latent Trajectory Studies Checklist (Table S3) (Van De Schoot et al., 2017).

This study represents a secondary analysis of data derived from an RCT, thus, the original trial was not designed nor powered for the present research question. Given the modest sample size, comparisons between treatment arms were considered beyond the scope of the present analysis and the findings should therefore be interpreted as exploratory. Nevertheless, simulation studies suggest that growth mixture modeling can be a suitable and robust approach for identifying longitudinal heterogeneity, even in relatively small samples (approximately 150 participants) (Martin and Von Oertzen, 2015).

The selection of the final model was based on a triangulation of the relative fit by Akaike's Information Criteria (AIC) and Bayesian Information Criteria (BIC), the classification accuracy by the Posterior Probability of Membership (PPM) and Entropy (indicator of the conditional probabilities of individuals' group membership) as well as by the size of the estimated classes and the substantive interpretability and clinical importance of the identified classes. There is no standardized way to identify the optimal result, but model selection should be informed by a variety of several criteria including theory, statistics and past findings (Ram and Grimm, 2009). Better model fit is indicated by lower AIC and BIC as well as higher PPM and entropy.

#### 2.5.2 Missing data handling and sensitivity analysis

The analysis was performed on subjects with at least 50% compliance (42 observations). In the raw format, 22.5% of data was missing. The moving-average procedure reduced missingness to 3%. Missing data was not imputed. While it is not possible to formally test whether data were missing at random (MAR) or missing not at random (MNAR), we examined potential patterns of missingness and did not observe systematic associations with baseline characteristics (Table S2), except for a weak correlation with age. A meta-analysis of pain EMA studies similarly reported that age and study duration were associated with missingness, whereas diagnosis and pain intensity were not (Ono et al., 2019).

Because restricting analyses to participants with higher compliance may introduce selection bias and may also affect the amount of missing data included in the analysis, we conducted a sensitivity analysis using a less conservative compliance threshold of 10% (minimum of 8 observations). This approach increases participant inclusion but allows a higher proportion of missing observations per individual. After preprocessing, this dataset contained approximately 20% missing data. Two outliers identified during data screening were removed prior to analysis. We further compared the full RCT sample with both subsamples (50% and 10% compliance) to examine potential selection bias based on baseline characteristics.

#### 2.5.3 Latent class contrasts

Chi-square tests and analysis of variance examined differences in baseline distributions across latent classes (*stats* and *rstatix* packages). Clinical effects according to changes in THI, TFI and PHQ-9 from baseline to 12-week final visit were calculated for each class and compared to Minimal Clinically Important Difference (MCID) estimates (THI = 11, TFI = 9, PHQ-9 = 6) (Hudgens et al., 2021; Engelke et al., 2025b). The distribution of classes within treatment arms is plotted only, as cell sizes do not allow for statistical testing. *P* values were *Holm-Bonferroni* adjusted for multiple comparisons, with the critical significance level set to *p* ≤ .05. All analyses were performed in R (Version 4.4.1).

#### 2.5.4 Trajectories of remaining symptoms

The trajectories of the remaining symptoms predicting clinical improvement were plotted as mean trajectories of pre-processed individual time-series (seven-day moving average) and 95% confidence intervals (CI). Based on that, we exploratively assessed the development of the association between daily tinnitus distress and maximum tinnitus loudness for each class with two additional analyses. The underlying assumption is that a reduction of the association can be considered as a desired treatment effect as tinnitus distress evolves to be evaluated more independent of tinnitus loudness (Engelke et al., 2023). We computed the weekly correlations between both symptoms for each class and the standardized difference between both symptoms ([d – mean{d}]/sd{d}; d = maximum tinnitus loudness - daily tinnitus distress) for each class. This quotient was standardized intra-individually. A value of 0 in the standardized quotient indicates the individual average difference between daily tinnitus distress and maximum tinnitus loudness. An increase in this quotient over time indicates that the difference between both becomes greater (desired treatment effect), while a decrease over time indicates that the difference becomes smaller (undesired treatment effect).

## 3 Results

### 3.1 Sample description

The sample consisted of 147 chronic tinnitus patients with 9634 observations (see Table 1). 45% were female, patients were on average 55 years old and 44% had graduated from university. The participants provided 65 observations on average (range: 42–84).

**Table 1:** Demographic and clinical characteristics at baseline.

Variable	Full sample (N = 147)	Class 1: deterioration	Class 2: stable course	Class 3: early improvement	Class 4: late improvement	p value
Gender						
Female	66 (44.9%)	12 (44.4%)	26 (44.1%)	16 (53.3%)	12 (38.7%)	0.730
Male	81 (55.1%)	15 (55.6%)	33 (55.9%)	14 (46.7%)	19 (61.3%)	0.730
Education						
Elementary school	16 (10.9%)	4 (14.8%)	4 (6.8%)	3 (10%)	5 (16.1%)	0.520
High school	23 (15.6%)	3 (11.1%)	9 (15.3%)	3 (10%)	8 (25.8%)	0.520
Middle school	44 (29.9%)	7 (25.9%)	18 (30.5%)	9 (30%)	10 (32.3%)	0.520
University or higher	64 (43.5%)	13 (48.1%)	28 (47.5%)	15 (50%)	8 (25.8%)	0.520
Age	54.72 (11.86)	58.78 (9.82)	54.36 (12.31)	55.63 (10.5)	51 (13.07)	0.090
THI score	49.17 (20.29)	49.56 (21.25)	46.71 (20.51)	51.4 (19.19)	51.35 (20.54)	0.660
TFI score	51.32 (21.23)	50.22 (22.89)	50.29 (19.93)	53.7 (20.19)	51.87 (23.8)	0.900
PHQ9 score	7.58 (4.93)	7.26 (5.35)	7.69 (4.99)	8.13 (5.24)	7.13 (4.28)	0.860
Hearing loss (PTA4)	20.61 (16.6)	19.07 (14.57)	20.53 (15.5)	21.72 (16.07)	21.02 (20.92)	0.940
Tinnitus duration (in months)	136.18 (120.98)	141.4 (123.25)	121.61 (123)	162.86 (136)	134.07 (99.39)	0.530
Extraversion	39.29 (6.84)	40.08 (7.94)	37.95 (6.55)	40.4 (7.08)	40.06 (6.01)	0.290
Agreeableness	47.18 (6.17)	48.04 (5.82)	46.52 (6.85)	48.13 (6.02)	46.77 (5.28)	0.570
Conscientiousness	46.94 (8.08)	45.38 (7.54)	47.21 (8.3)	48.47 (8.31)	46.29 (7.98)	0.520
Neuroticism	34.19 (8.27)	34.58 (8.24)	35.26 (7.94)	33.33 (9.45)	32.71 (7.76)	0.510
Openness	43.72 (7.48)	42.65 (7.95)	42.91 (8.58)	45.07 (6.34)	44.84 (5.62)	0.420
Number of comorbidities	2.28 (2.14)	2.89 (2.82)	2.15 (2.12)	1.97 (1.71)	2.29 (1.88)	0.390
Number of tinnitus treatments	0.32 (0.64)	0.41 (0.8)	0.37 (0.67)	0.13 (0.51)	0.32 (0.54)	0.330

*Notes*. Chi-square tests and analysis of variance examined differences in baseline distributions across latent classes. All Holm-Bonferroni adjusted *p*-values were equal to 1. THI (Tinnitus Handicap Inventory) score range: 0–100. TFI (Tinnitus Functional Index) score range: 0–100. PHQ-9 (Patient Health Questionnaire-9) score range: 0–27. PTA4: Average hearing threshold at the frequencies 500 Hz, 1 kHz, 2 kHz and 4 kHz. Top five comorbidities: sleep difficulties (N = 37), high blood pressure (N = 26), high cholesterol (N = 26), acid reflux (N = 24), thyroid disorder (N = 21). Number of tinnitus treatments received prior to study participation. BFI-2 (Big Five Inventory-2) dimensions score range: 12–60.

### 3.2 Model selection

All spline models had a better relative fit than their linear equivalents (Fig. S2). The relative fit of the spline models corresponded to an asymptotic reduction of AIC and BIC with increasing number of classes without a reasonable minimum indicating a clearly favorable class solution (Fig. S2b). Thus, more weight was put on the other metrics. As comparable literature identified three-, four- and five-class solutions (Ulvenes et al., 2022; Saunders et al., 2019; Espel-Huynh et al., 2020), these solutions were more closely inspected (Fig. S3). PPM and entropy values were high for all three models with a slight tendency towards the four-class solution (Table S4). Inspecting the trajectories, class 4 in the four-class model adds an interesting and potentially relevant group describing a later-onset response. In the five-class model, the new trajectory is captured by class 2, however, the relevance of its distinction from class 3 is ambiguous as neither one describes an improvement (Fig. S3c). Further, class 2 is small (10.9%, N = 16) and CIs overlap strongly in the five-class solution. Thus, to remain conservative and avoid overfitting, we decided that the four-class latent trajectories describe the data best.

### 3.3 Description of latent trajectories

**Fig. 1:**
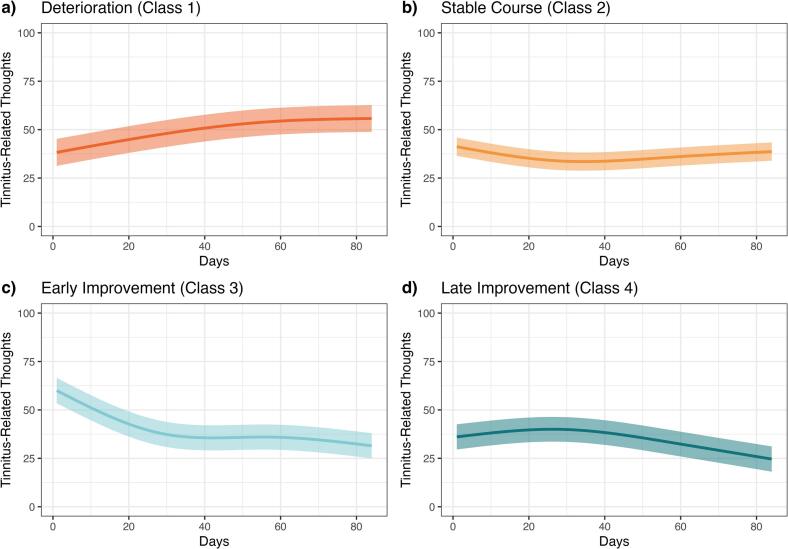
Latent trajectories of the final model *Notes.* The four-class model was chosen due to its highest entropy and PPM values as well as because class 2 in the five-class model was considered irrelevant from a size and clinical point of view (Fig. S3). Trajectories depict estimated values, transparent bars are 95% CIs.

The final result consisted of four classes with distinct trajectories (Fig. 1). Class 1 depicted increasing tinnitus-related thoughts and was labelled *Deterioration*. (18.4%, N = 27). The estimated trajectory started at 38 (95% CI: 31–45) and showed an increase to 56 (95% CI: 49–63). Class 2 patients showed no change in the outcome and was labelled *Stable Course* (40.1%, N = 59). The trajectory decreased slightly from 41 (95% CI: 37–46) on the first day until 34 (95% CI: 29–38) on day 34 before it increased back to 39 (95% CI: 34–43). Class 3 showed an early response in tinnitus-related thoughts and was labelled *Early Improvement* (20.4%, N = 30). The trajectory depicted the highest baseline of 60 (95% CI: 54–67) and a strong decrease during the first half of the treatment period until 36 (95% CI: 29–42) on day 42 followed by a short counter climb before it decreased again to 32 (95% CI: 25–38). Finally, group 4 (21.1%, N = 31) showed a late-onset response and was labelled *Late Improvement*. The estimation started at 36 (95% CI: 30–43), showed a small increase until 40 (95% CI: 34–46) on day 26 before it decreased to 25 (95% CI: 18–31). The individual observed trajectories are plotted in Fig. S4.

### 3.4 Sensitivity analysis

The latent trajectories of the four-class result based on the 10% compliance data set are depicted in Fig. S5b. Deterioration featured 22%, Stable Course 36%, Early Improvement 15%, and Late Improvement 27% of participants. PPM was high (0.94–0.99) and entropy was acceptable (0.88). Trajectories differed minimally from the four-class result based on the 50% compliance sample (see Fig. S5a). Both subsets (10% and 50% compliance) were compared with the full RCT sample based on baseline characteristics, only age showed significant differences between samples (see Table S5).

### 3.5 Latent class contrasts

Demographic or clinical variables at baseline were not predictive of latent trajectory membership (see Table 1).

**Fig. 2:**
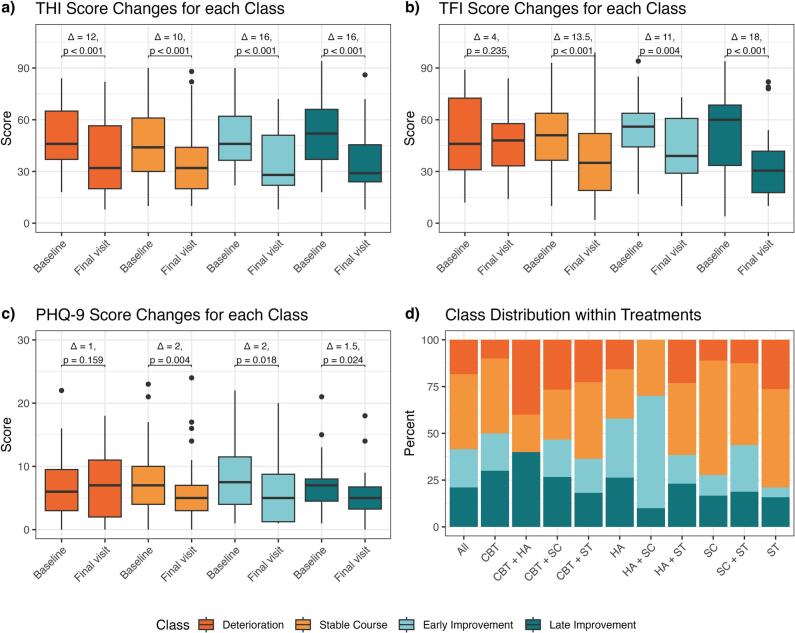
PROM changes and treatments across classes *Notes.* Figures a-c depict PROM changes across classes. THI (Tinnitus Handicap Inventory) score range: 0–100. TFI (Tinnitus Functional Index) score range: 0–100. PHQ-9 (Patient Health Questionnaire-9) score range: 0–27. Figure d depicts the distribution of class size for each treatment arm. Treatments were provided single or in combination: Cognitive Behavioural Therapy (CBT), Hearing Aids (HA), Structured Counseling (SC), and Sound Therapy (ST).

Fig. 2a–c depicts relevant PROM changes across classes. THI score improved significantly for each class from baseline to final visit with clinically relevant improvements for Deterioration, Early and Late Improvement. TFI score improved significantly and clinically relevant for Stable Course, Early and Late Improvement. The PHQ-9 improved significantly for Stable Course, Early and Late Improvement, none reached clinically significance. The distribution of class size for each treatment arm is depicted in Fig. 2d.

### 3.6 Trajectories of remaining symptoms

The trajectories of the remaining symptoms relevant for clinical improvement (jaw tension, momentary tinnitus loudness, daily tinnitus distress, maximum tinnitus loudness, and negative emotion) are depicted for each class in Fig. S6. Daily tinnitus distress varied very closely with tinnitus-related thoughts in each class. Maximum and daily tinnitus loudness was rated the highest across time and classes, jaw tension is rated the weakest. Trends in the tinnitus-related thoughts, if available, are reflected less pronounced in some symptoms and classes.

**Fig. 3:**
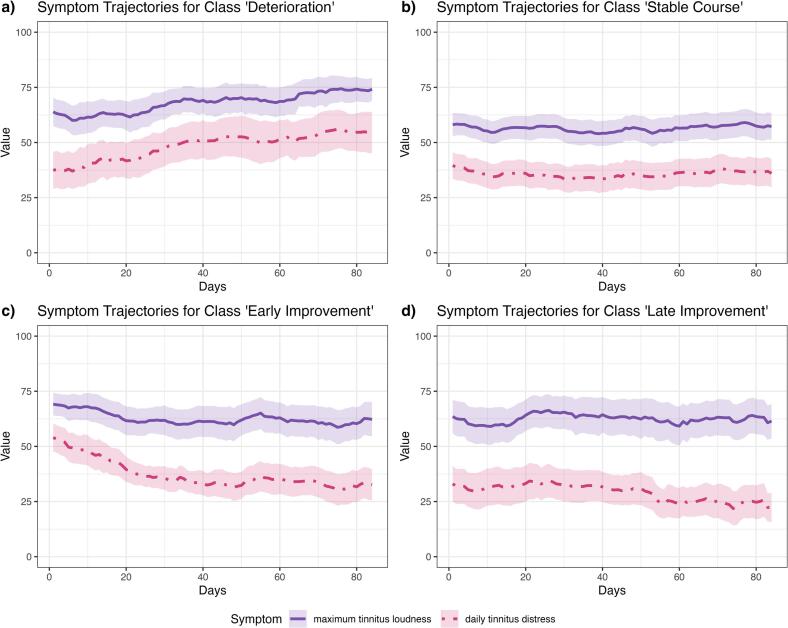
Trajectories of tinnitus loudness and distress across classes *Notes.* Trajectories are mean values of pre-processed individual time-series (seven-day moving average), transparent bars are 95% CIs. All symptoms were rated on a visual analogue scale from 0 to 100.

**Fig. 4:**
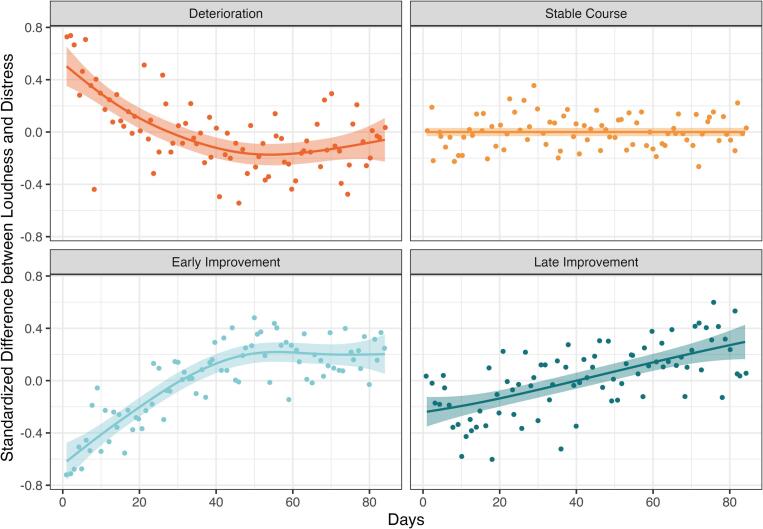
Distribution of the intra-individual standardized difference (maximum tinnitus loudness – daily tinnitus distress) by class. *Note.* Distribution of the intra-individual standardized difference between maximum tinnitus loudness and daily tinnitus distress ([d – mean{d}]/sd{d}; d = maximum tinnitus loudness – daily tinnitus distress). Dots represent mean values by class and day. Trend lines depict the smoothed trend and 95% CI (geom_smooth in ggplot). Intra-individual standardization means that positive values indicate a greater difference between maximum tinnitus loudness and daily tinnitus distress compared to the individual's average difference, while negative values indicate a smaller difference compared to their individual average. An increase in this quotient over time indicates that the difference between both becomes greater (desired treatment effect), while a decrease over time indicates that the difference becomes smaller (undesired treatment effect).

Fig. 3 shows the mean trajectories of daily tinnitus distress and maximum tinnitus loudness. The weekly average correlation between both remained descriptively stable for the classes Deterioration and Stable Course and decreased for the classes Early and Late Improvement (Fig. S7). When looking at the standardized difference between daily tinnitus distress and maximum tinnitus loudness, the difference decreased in the Deterioration Class, remained stable in the Stable Course Class, and increased in both the Early and Late Improvement Classes (Fig. 4).

## 4 Discussion

The present study demonstrated that EMA-measured trajectories in tinnitus-related thoughts while patients received established interventions can be described by four distinct trajectories. Roughly one-fifth of participants showed a progressive increase (Deterioration, 18%), two-fifths experienced no discernible change (Stable Course, 40%), while the remaining patients split almost evenly between an early response (Early Improvement, 20%) and a late-onset response pattern in tinnitus-related thoughts (Late Improvement, 21%). Despite different metrics and treatment formats used, this is in line with Nieman and colleagues (Niemann et al., 2024). Both studies converge on the existence of multiple distinct symptom-change subgroups, prospectively demonstrating heterogeneity in treatment response.

Similar research in mental health has mostly identified three- to four-class solutions, suggesting that the responder/non-responder dichotomy masks clinically relevant heterogeneity (Ulvenes et al., 2022; Saunders et al., 2019; Espel-Huynh et al., 2020). Our finding of early and late responders aligns with prior evidence that improvement can follow different pacing, sometimes with temporary deterioration before benefits emerge (Saunders et al., 2019; Espel-Huynh et al., 2020). Such initial worsening may reflect psychological activation processes (Lin et al., 2024) and, in tinnitus, increased symptom vigilance during treatment. Communicating this to patients may help manage expectations and reduce dropout, as seen in the “Late Improvement” group (Table S6).

Applying a 50% EMA completion threshold excluded many participants, but replication with a 10% threshold produced nearly identical class trajectories and proportions. Both subsets closely matched the full RCT sample (except for minor age differences), suggesting minimal selection bias. Importantly, this remains a key limitation of EMA data. The sensitivity analysis highlights the inherent trade-off in real-world EMA studies with missing data: either restricting analyses to a smaller subset with higher compliance or including a larger sample with greater missingness. This may introduce either bias or uncertainty, and the results should therefore be interpreted with caution.

Baseline demographics and clinical data showed no significant class differences, suggesting that generic prediction of treatment response is difficult and highlighting heterogeneity in treatment needs. However, given the modest sample size, the absence of significant predictors was expected. Also, random allocation likely resulted in some patients receiving optimal and others sub-optimal interventions, consistent with the presence of all trajectories across treatment arms. Nonetheless, predicting response to specific treatments based on baseline characteristics remains possible. Precision matching approaches, such as the Personalized Advantage Index (PAI), may help optimize treatment allocation using prescriptive predictors (DeRubeis et al., 2014).

Growth mixture modeling of EMA data revealed subgroups with stable or worsening tinnitus-related thoughts, whereas changes in PROMs indicated that more groups/patients benefited from treatment. The “Stable Course” class improved clinically in the TFI, and the “Deterioration” class improved clinically in the THI score. Further exploration of this discrepancy is highly important, as it may indicate an overestimation of treatment effects by PROMs or an underestimation by EMA data. Psychological research conceptualizes this difference as due to an “experiencing self” and “remembering self”, which represent distinct modes of evaluating experiences (Kahneman and Riis, 2005). In this context, the memory of an experience does not simply reflect the mean of the whole period but rather is disproportionately influenced by the peak and the end of the period (peak/end rule) (Kahneman and Riis, 2005). It remains an open question which processes are more important for understanding patient improvement and well-being.

Average trajectories showed that tinnitus distress closely paralleled tinnitus-related thoughts, suggesting one construct may suffice to reduce burden without loss of information. In contrast, loudness remained high across classes, supporting its partial independence from distress (Ueyama et al., 2013; Wallhäußer-Franke et al., 2012). Correlation analyses and trajectories of standardized difference indicated stable loudness–distress associations in “Deterioration” and “Stable Course,” but divergence in “Early” and “Late Improvement,” potentially reflecting treatment success (Engelke et al., 2023). This pattern also suggests current treatments mainly reduce burden rather than loudness, with early improvers plateauing around day 50, while late improvers continued to diverge, possibly with further gains under longer treatment.

The following limitations should be considered. First, we hypothesize that improvements of 28 and 11 tinnitus-related thoughts points in both response groups are clinically relevant, yet, the available estimation of clinically relevant differences is based on tinnitus distress and loudness (Adamchic et al., 2012). Second, daily-measured tinnitus-related thoughts is no established outcome measure even if it revealed some predictive power in an earlier investigation (Engelke et al., 2025b). Third, our analysis relied on a subsample, posing potential selection bias despite sensitivity analyses and comparisons with the full sample. Smartphone-based data collection and limited sample size add to this risk. Although moving averages reduced missingness, future studies should prioritize maximizing compliance to minimize data loss. As outlined before, this is remains a critical challenge in EMA research (Drexl et al., 2025; Stone et al., 2023). Fourth, growth mixture models require a lot of choices that could influence the number and shape of trajectories. By adhering to the reporting guidelines, we followed the currently existing standard procedure. Fifth, the absence of a clear AIC/BIC minimum suggests that the underlying heterogeneity may be more continuous than categorical. Accordingly, the identified classes should be interpreted with caution, as they may represent convenient approximations of a gradient rather than truly distinct subgroups. At the same time, such models provide a useful framework for summarizing complex patterns of heterogeneity in an interpretable way. A larger sample size may help to further clarify this structure. Sixth, due to the large number of treatment arms, our sample lacked power to search for class-by-treatment interactions.

Despite limitations, our findings reveal substantial heterogeneity in symptom trajectories, opening avenues for research and practice. The present study highlights several lessons for the future use of EMA in this field. First, careful consideration should be given to the number and timing of assessments to balance data richness with participant burden, as overly intensive sampling may increase missingness. Identifying a core set of clinically informative items may help optimize this balance by focusing on the most relevant symptoms while reducing response fatigue. Second, the observed heterogeneity in trajectories underscores the value of repeated, high-frequency assessments beyond traditional pre–post designs, as important changes may otherwise remain undetected. Third, future studies should examine whether the four trajectory groups identified here can be replicated in independent samples and treatment settings. Replication of both the number of classes and their course over time will be essential for determining the robustness and clinical relevance of these patterns.

More broadly, intensive monitoring can yield valuable insights, ideally complemented by passive data to reduce burden. Precision medicine, the data-driven attempt to individualise treatment, strongly benefits from continuous monitoring of symptom trajectories to regularly check if patients are “on track” and adapt treatment plans to individual needs based on real-time data (Delgadillo and Lutz, 2026). Together with parallel efforts in prescriptive research initial allocation of patients to treatments could be improved, while continued monitoring beyond treatment may clarify whether improvements consolidate, fade, or require maintenance strategies.

In summary, high-intensive longitudinal monitoring reveals rich, non-linear patterns of change that remain invisible to traditional assessment schedules. Embracing this complexity will be essential to tailor interventions to inter- and intraindividual needs to ultimately alleviate the burden of tinnitus for the diverse population of sufferers.

## CRediT authorship contribution statement

**Milena Engelke:** Conceptualization, Methodology, Formal analysis, Writing – original draft, Visualization. **Berthold Langguth:** Writing – review & editing, Supervision. **Thomas Probst:** Writing – review & editing, Supervision. **Rüdiger Pryss:** Writing – review & editing. **Stefan Schoisswohl:** Writing – review & editing, Project administration, Investigation. **Jorge Piano Simões:** Writing – review & editing, Methodology. **Peter Ten Klooster:** Writing – review & editing, Methodology. **Beat Toedtli:** Writing – review & editing. **Carsten Vogel:** Writing – review & editing. **Winfried Schlee:** Conceptualization, Writing – review & editing, Supervision, Project administration, Investigation, Funding acquisition.

## Declaration of generative AI and AI-assisted technologies in the writing process

During the preparation of this work the authors used ChatGPT for copy-assisted editing. After using this tool, the authors reviewed and edited the content as needed and take full responsibility for the content of the publication.

## Declaration of competing interest

W.S. and R.P. are stakeholders of the Lenox UG but declare no non-financial competing interests. The Lenox UG aims to translate scientific knowledge into e-health applications and also holds shares of the HealthStudyClub GmbH. W.S. received consulting fees as well as payments for lectures in connection with chronic tinnitus topics but declared no non-financial competing interests. R.P. received consulting fees, reimbursements for congress attendance, and travel expenses as well as payments for lectures in connection with mobile health and e-mental health topics but declares no non-financial competing interests. R.P. serves as Associate Editor of this journal and had no role in the peer review or decision to publish this manuscript. All other authors declare no financial or non-financial competing interests.

## Data Availability

The data is available from the corresponding author upon reasonable request.
