# Supplementary material for: Uncovering latent trajectories of daily tinnitus symptoms through app-based monitoring during treatment
Source: Internet Interv. 2026 Apr 8;44:100943. doi: 10.1016/j.invent.2026.100943 (PMC13091948; doi:10.1016/j.invent.2026.100943)
Supplement: Supplementary file 1 — Supplementary material [file mmc1.docx]

**Table S1. Daily measured tinnitus and health-related symptoms.**

|  | **Symptom** | **Question** | **VAS scale [lowest anchor - highest anchor]** |
| --- | --- | --- | --- |
| 1. | Momentary tinnitus distress | How burdensome do you find your tinnitus at the moment? | not burdensome –  very burdensome |
| 2. | Daily tinnitus distress | To what extent did you feel affected by the tinnitus today? | not at all –  the whole day |
| 3. | Momentary tinnitus loudness | How loud is your tinnitus at the moment? | inaudible –  very loud |
| 4. | Maximum tinnitus loudness | What was the maximum tinnitus volume today? | inaudible –  very loud |
| 5. | Tinnitus-related thoughts | How often have you thought about tinnitus today? | not at all –  the whole day |
| 6. | Emotion | What emotion would you use to describe today? | [sad emoji] –  [happy emoji] |
| 7. | Jaw tension | How tense does your jaw feel right now? | not at all tense –  very tense |
| 8. | Movement | How much did you move today? | not at all –  very much |
| 9. | Neck tension | How tense does your neck feel right now? | not at all tense –  very tense |
| 10. | Stress | How stressed did you feel today? | not at all stressed –  very stressed |

*Note*. Questions were answered on a continuous visual analogue scale ranging from 0-100 (numbers were invisible to the patient).

**Figure S1. Distribution of tinnitus-related thoughts.**


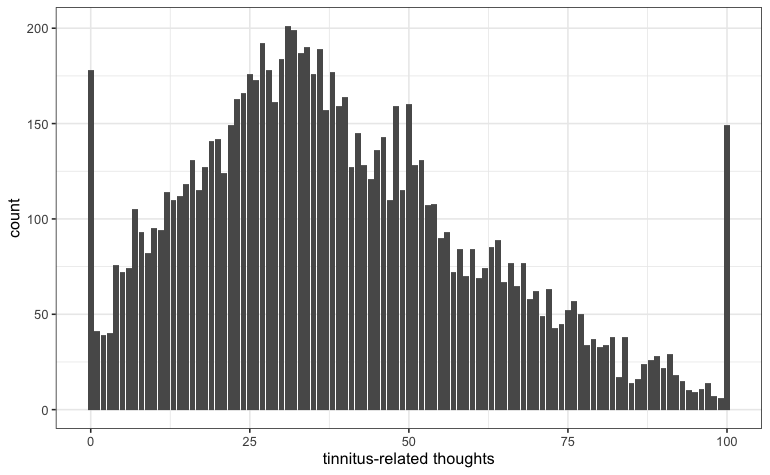


| **Table S2. Correlation with missingness** | | | |
| --- | --- | --- | --- |
| **variable** | **correlation** | **p value** | **adjusted p value** |
| CGI-I | 0.06 | 0.46 | 1.00 |
| THI score baseline | 0.10 | 0.25 | 1.00 |
| THI score final visit | 0.02 | 0.85 | 1.00 |
| TFI score baseline | 0.03 | 0.74 | 1.00 |
| TFI score final visit | -0.06 | 0.50 | 1.00 |
| PHQ9 score baseline | 0.05 | 0.52 | 1.00 |
| PHQ9 score final visit | 0.09 | 0.30 | 1.00 |
| Age | -0.26 | 0.00 | 0.02 |
| Extraversion | 0.02 | 0.78 | 1.00 |
| Agreeableness | -0.06 | 0.47 | 1.00 |
| Conscientiousness | -0.02 | 0.85 | 1.00 |
| Neuroticism | 0.06 | 0.46 | 1.00 |
| Openness | -0.01 | 0.86 | 1.00 |
| Hearing loss (PTA4) | -0.03 | 0.70 | 1.00 |
| *Note.* Correlation of patient characteristics with amount of missingness in EMA data to check for a systematic pattern of missingness. P values were corrected according to Holm. | | | |

**Table S3. Guidelines for Reporting on Latent Trajectory Studies (GRoLTS) Checklist**

| Checklist item | reported |
| --- | --- |
| 1. Is the metric of time used in the statistical model reported? | yes |
| 2. Is information presented about the mean and variance of time within a wave? | yes |
| 3a. Is the missing data mechanism reported? | yes |
| 3b. Is a description provided of what variables are related to attrition/missing data? | yes |
| 3c. Is a Description Provided for How Missing Data Were Handled in the Analyses? | yes |
| 4. Is information about the distribution of the observed variables included? | yes |
| 5. Is the software mentioned? | yes |
| 6a. Are alternative specifications of within-class heterogeneity considered (e.g., LGCA vs. LGMM) and clearly documented? If not, was sufficient justification provided as to eliminate certain specifications from consideration? | yes |
| 6b. Are Alternative Specifications of the BetweenClass Differences in Variance–Covariance Matrix Structure Considered and Clearly Documented? | yes |
| 7. Are Alternative Shape and Functional Forms of the Trajectories Described? | yes |
| 8. If Covariates Have Been Used, Can Analyses Still Be Replicated? | yes |
| 9. Is Information Reported About the Number of Random Start Values and Final Iterations Included? | yes |
| 10. Are the model comparison (and selection) tools described from a statistical perspective? | yes |
| 11. Are the total number of fitted models reported, including a one-class solution? | yes |
| 12. Are the number of cases per class reported for each model (absolute sample size, or proportion)? | yes |
| 13. If classification of cases in a trajectory is the goal, is entropy reported? | yes |
| 14a. Is a plot included with the estimated mean trajectories of the final solution? | yes |
| 14b. Are plots included with the estimated mean trajectories for each model? | yes |
| 14c. Is a plot included of the combination of estimated means of the final model and the observed individual trajectories split out for each latent class? | yes |
| 15. Are characteristics of the final class solution numerically described (i.e., means, SD/SE, n, CI, etc.)? | yes |
| 16. Are the syntax files available (either in the appendix, supplementary materials, or from the authors)? | yes |

**Figure S2. Relative Fit of Growth Mixture Models.**


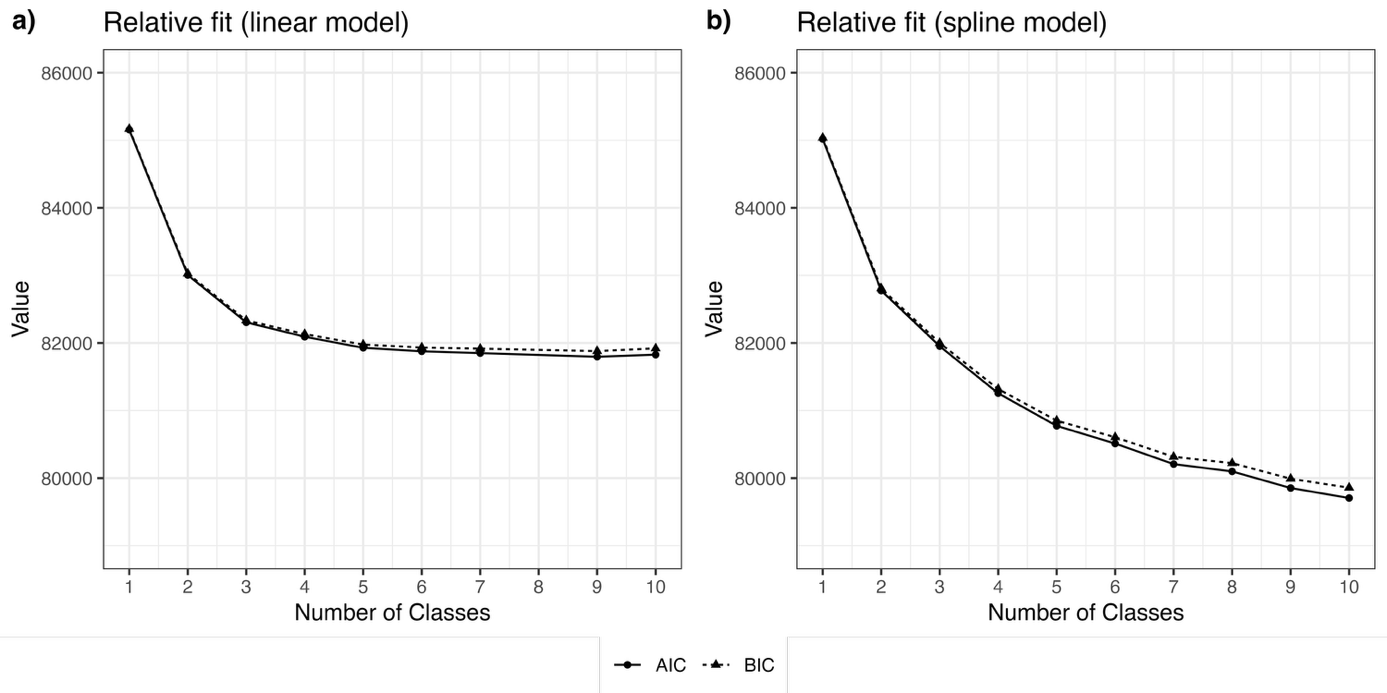


*Notes.* Better relative fit of spline models than linear models. Due to asymptotic reduction of AIC and BIC among spline models, more weight was put on clinical interpretability in the selection of the final model. The linear eight-class model did not converge.

**Figure S3. Model Selection Based on Clinical Interpretability.**


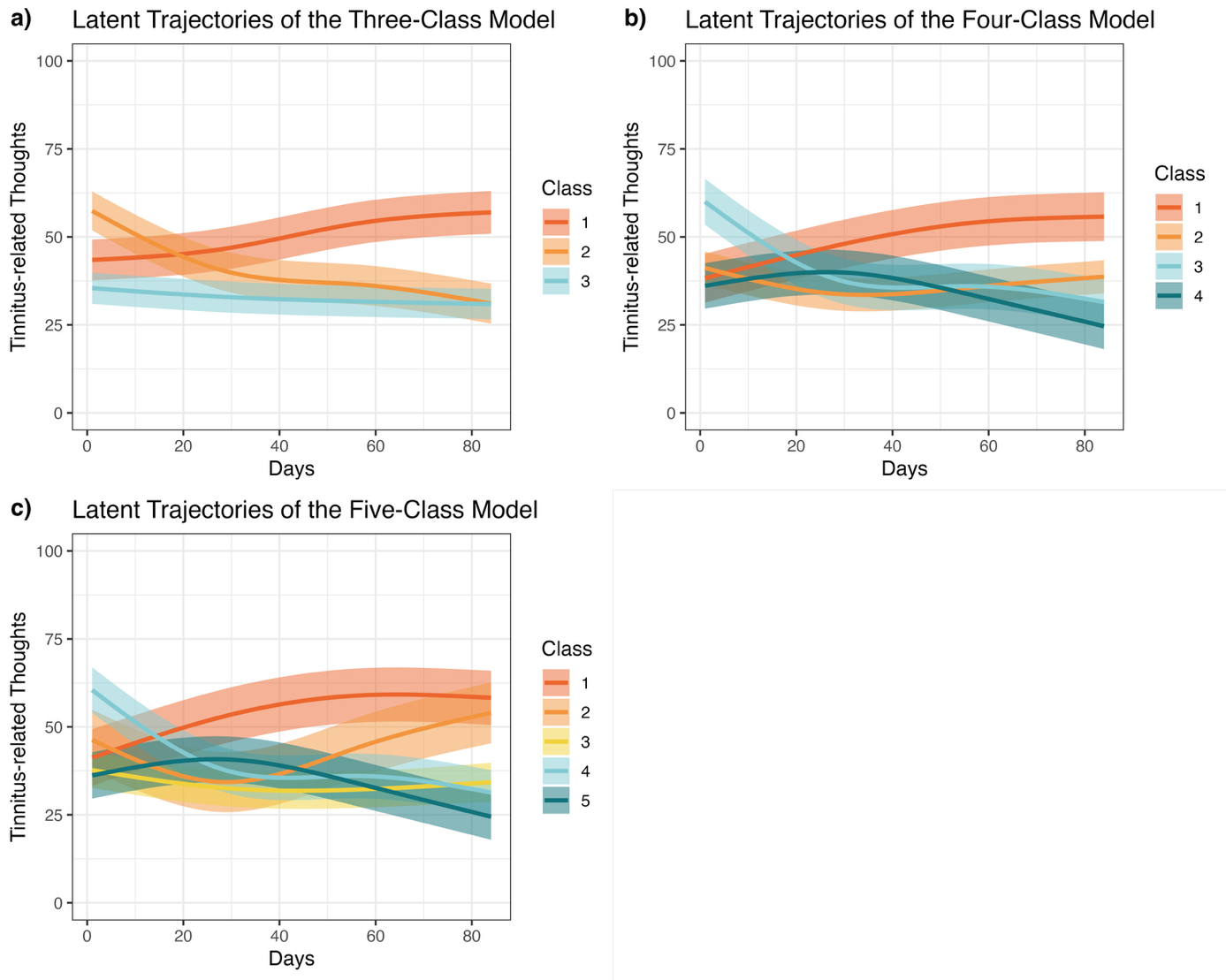


*Notes.* Trajectories depict estimated values, transparent bars are 95% CIs. Results were derived from growth mixture analysis estimating trajectories of tinnitus-related thoughts during treatment with varying number of latent classes.

**Table S4. Model Selection Based on Entropy, Class Size, and PPM**

| **Model** | **Entropy** | **Class Size** | | **Posterior Probability of Membership** | |
| --- | --- | --- | --- | --- | --- |
| Three-class | 0.91 | Class 1 | 25.9% | Class 1 | 0.98 |
|  |  | Class 2 | 27.2% | Class 2 | 0.98 |
|  |  | Class 3 | 46.9% | Class 3 | 0.98 |
| Four-class | 0.97 | Class 1 | 18.4% | Class 1 | >0.99 |
|  |  | Class 2 | 40.1% | Class 2 | 0.99 |
|  |  | Class 3 | 20.4% | Class 3 | >0.99 |
|  |  | Class 4 | 21.1% | Class 4 | 0.99 |
| Five-class | 0.96 | Class 1 | 15.0% | Class 1 | >0.99 |
|  |  | Class 2 | 10.9% | Class 2 | 0.99 |
|  |  | Class 3 | 35.4% | Class 3 | 0.98 |
|  |  | Class 4 | 19.7% | Class 4 | 0.99 |
|  |  | Class 5 | 19.1% | Class 5 | 0.99 |

*Notes.* Models were derived from growth mixture analysis estimating trajectories of tinnitus-related thoughts during treatment with varying number of latent classes.

**Figure S4. Observed Trajectories for Each Class.**


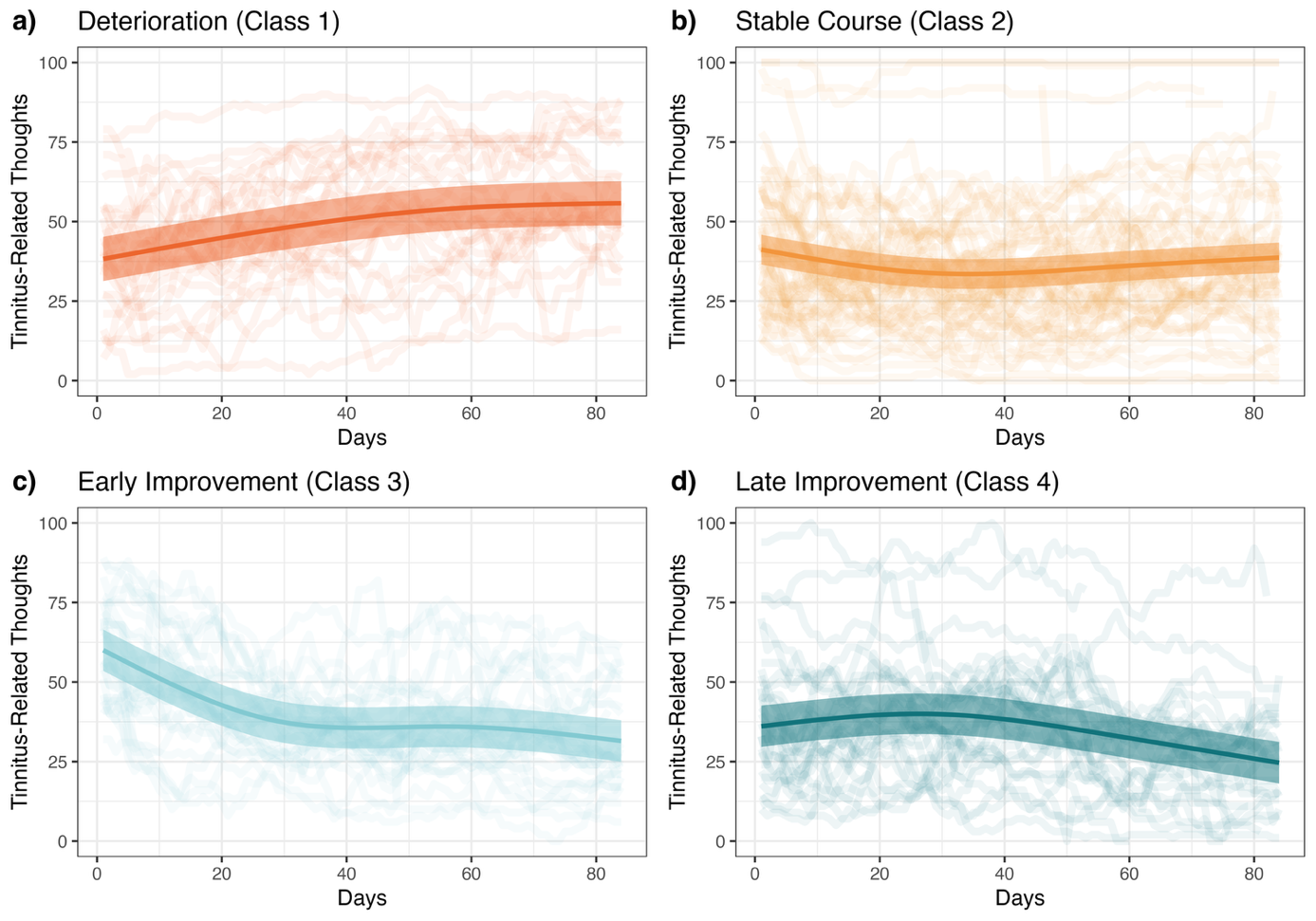


*Note*. The bold trajectories depict estimated values, transparent bars are 95% CIs. The transparent lines depict the observed trajectories.

**Figure S5. Latent Trajectories of the Sensitivity Analysis**


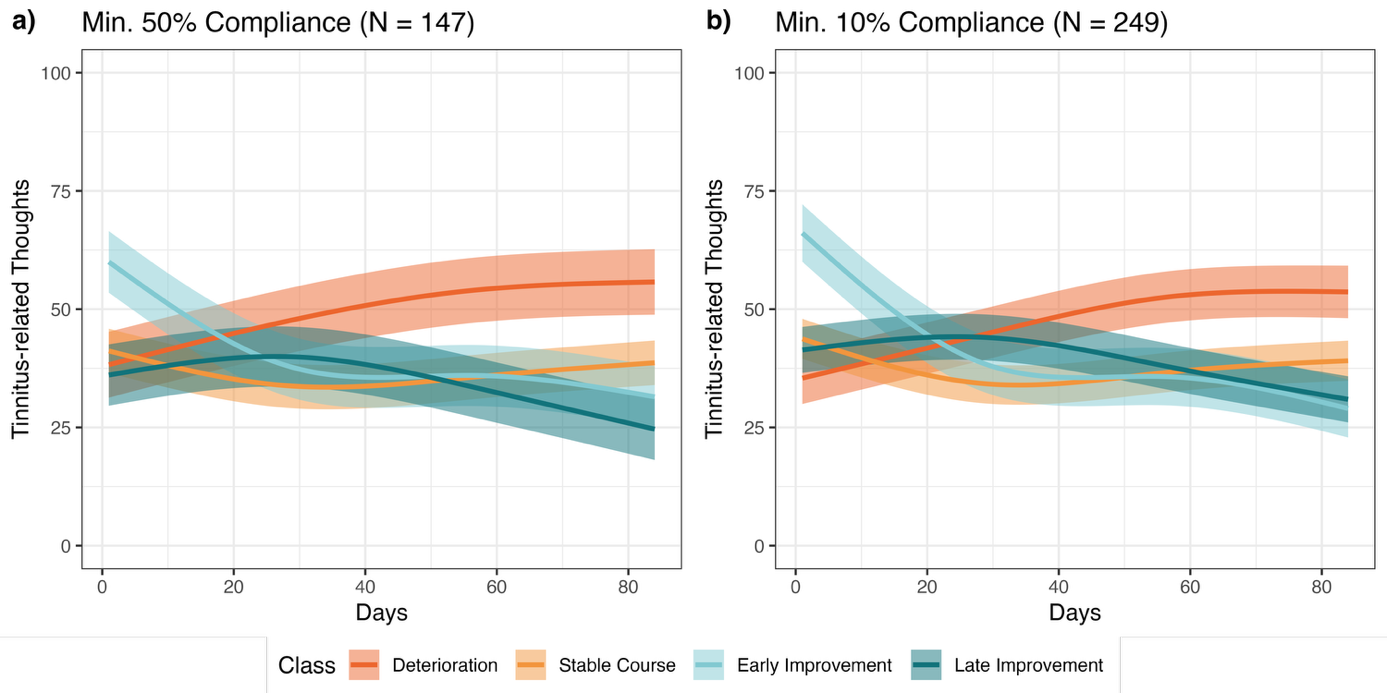


*Notes.* Comparison of results of the growth mixture analysis with two different cut-off values regarding compliance. The analysis in a) included participants with at least 50% compliance to the EMA protocol, the analysis in b) included participants with at least 10% compliance to the EMA protocol. Trajectories depict estimated values, transparent bars are 95% CIs.

**Table S5. Comparison of subsamples to the RCT sample on baseline characteristics.**

| **Baseline Characteristics** | **10% compliance** | **50% compliance** | **RCT sample** | ***p value*** | ***adjusted p value*** |
| --- | --- | --- | --- | --- | --- |
| Gender |  |  |  |  |  |
| female | 110 (44.2%) | 66 (44.9%) | 195 (40.8%) | .540 | 1.00 |
| male | 139 (55.8%) | 81 (55.1%) | 283 (59.2%) | .540 | 1.00 |
| Age | 51.92 (12.66) | 54.72 (11.86) | 50.95 (12.42) | .010* | 0.07 |
| THI score | 49.05 (20.06) | 49.17 (20.29) | 48.02 (19.73) | .730 | 1.00 |
| PHQ9 score | 7.62 (5.19) | 7.58 (4.93) | 7.35 (4.87) | .750 | 1.00 |
| Hearing loss (PTA4) | 19.28 (15.98) | 20.61 (16.6) | 19.21 (15.38) | .630 | 1.00 |
| Tinnitus duration (in months) | 124.33 (113.61) | 136.18 (120.98) | 119.48 (113.37) | .330 | 1.00 |
| Extraversion | 40.37 (7.25) | 39.29 (6.84) | 40.6 (7.53) | .170 | 1.00 |
| Agreeableness | 47.51 (6.18) | 47.18 (6.17) | 47.1 (5.97) | .700 | 1.00 |
| Conscientiousness | 47.06 (8.16) | 46.94 (8.08) | 47.19 (7.94) | .940 | 1.00 |
| Neuroticism | 34.4 (8.16) | 34.19 (8.27) | 34.85 (8.07) | .630 | 1.00 |
| Openness | 44.35 (7.6) | 43.72 (7.48) | 44.49 (7.63) | .580 | 1.00 |

*Note*. THI (Tinnitus Handicap Inventory) score range: 0-100. PHQ-9 (Patient Health Questionnaire-9) score range: 0-27. PTA4: Average hearing threshold at the frequencies 500 Hz, 1 kHz, 2 kHz and 4 kHz. BFI-2 (Big Five Inventory-2) dimensions score range: 12-60.

**Figure S6. Trajectories of Remaining Symptoms Across Classes**


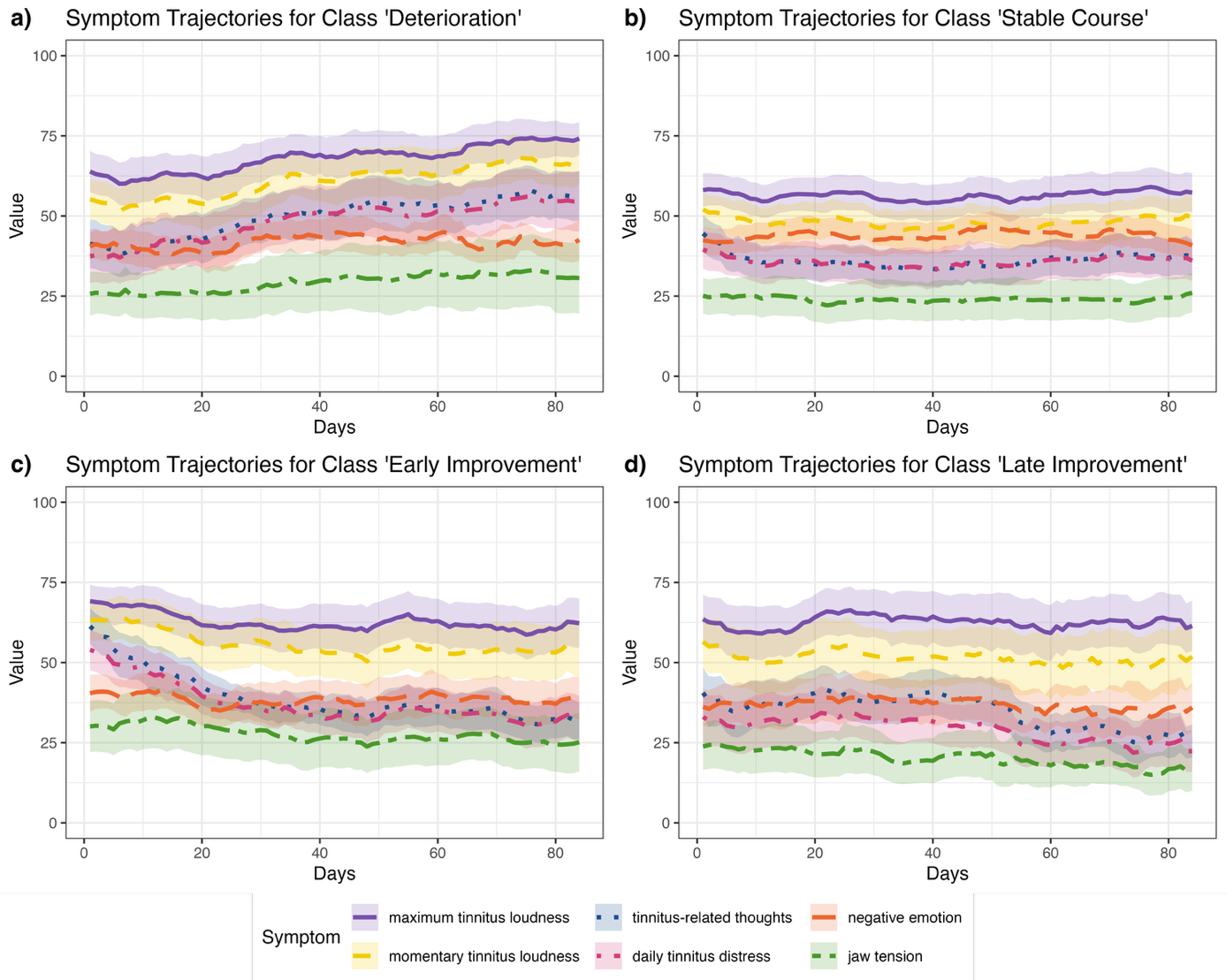


*Notes.* Trajectories are mean values of pre-processed individual time-series (seven-day moving average), transparent bars are 95% CIs. All symptoms were rated on a visual analogue scale from 0 — 100.

**Figure S7. Correlation of maximum tinnitus loudness with daily tinnitus distress**


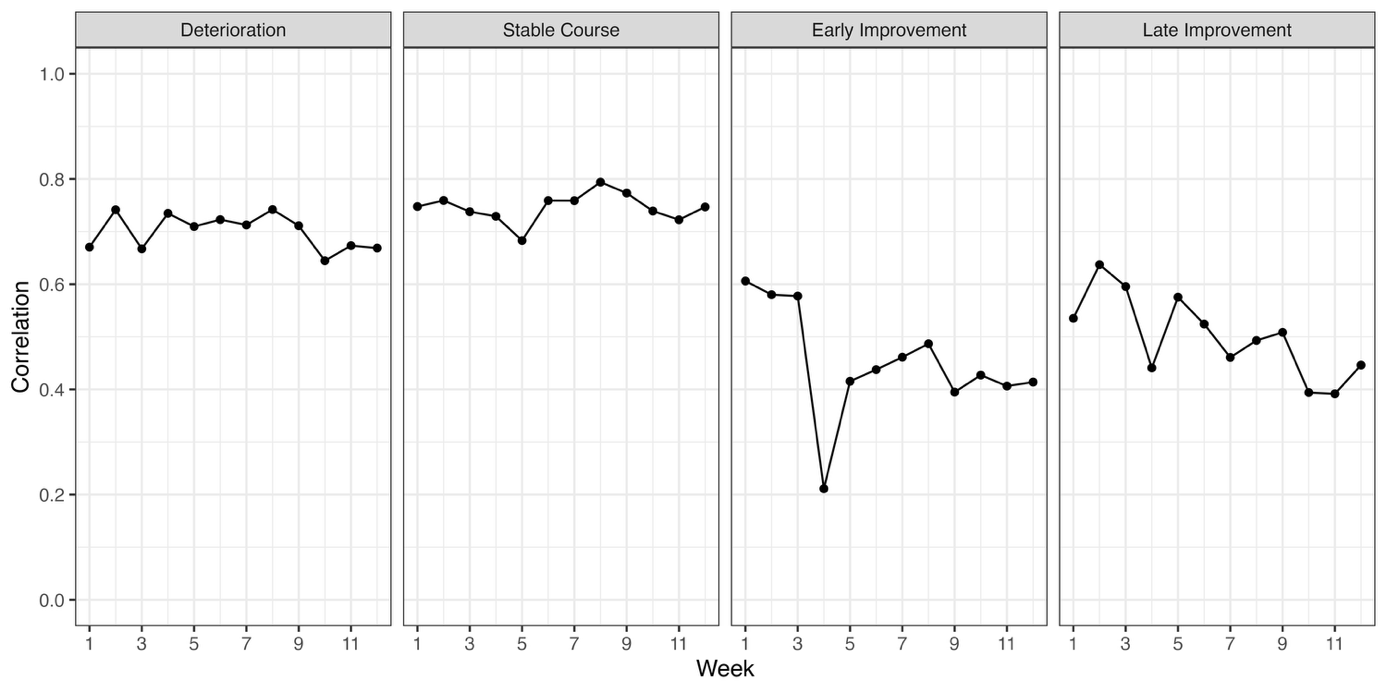


*Note*. Dots depict weekly average correlations between maximum tinnitus loudness and daily tinnitus distress (raw values) for each class.

**Table S6. Dropouts and discontinuation during treatment by class.**

| **Class** | **N** | **%** |
| --- | --- | --- |
| Deterioration | 4 | 14.8 |
| Stable Course | 5 | 8.5 |
| Early Improvement | 1 | 3.3 |
| Late Improvement | 5 | 16.1 |
